# Supplementary material for: Candidemia among Hospitalized Pediatric Patients Caused by Several Clonal Lineages of Candida parapsilosis
Source: J Fungi (Basel). 2022 Feb 12;8(2):183. doi: 10.3390/jof8020183 (PMC8880282; doi:10.3390/jof8020183)
Supplement: Supplementary file 1 [file jof-08-00183-s001.zip › final Cparapsilosis typing - supp Figures.pdf]

Supplementary Figures

Figure S1. AFLP fingerprinting of included isolates.

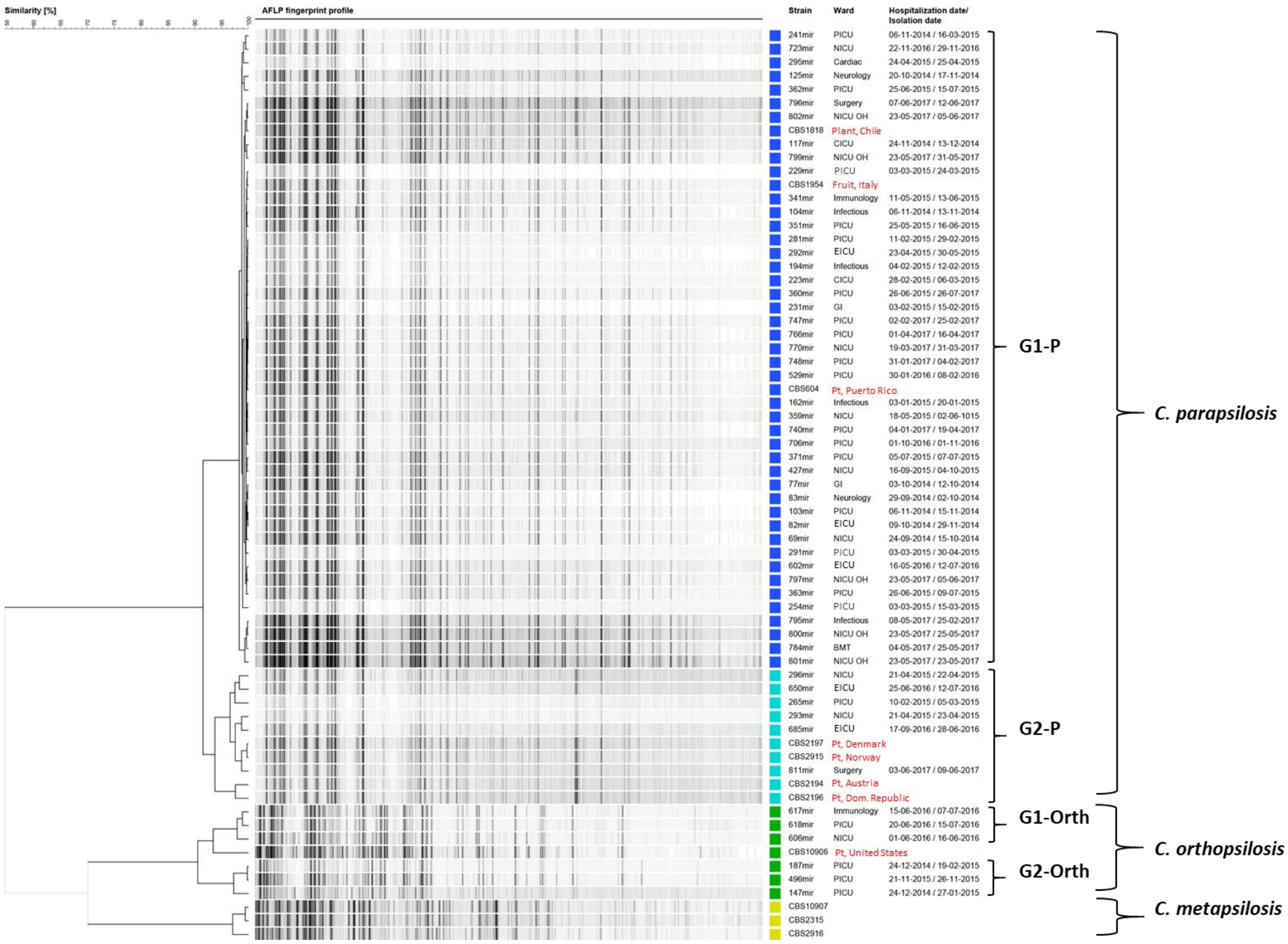

Figure S1. Amplified Fragment Length Polymorphism (AFLP) fingerprinting of *C. parapsilosis* complex species [1]. Colors indicate the different AFLP clusters, G1-P and G2-P for *C. parapsilosis* and G1-Orth and G2-Orth for *C. orthopsilosis,* respectively. Reference strains (CBS) are included and origins are provided in red text. Pt: patient, ICU: Intensive care unit, PICU: Pediatric ICU, NICU: Neonatal ICU, NICU-OH: NICU Open Heart Surgery, CICU: Cardiac ICU, EICU: Emergency ICU, GI: Gastroenterology, BMT: Bone marrow transplant.

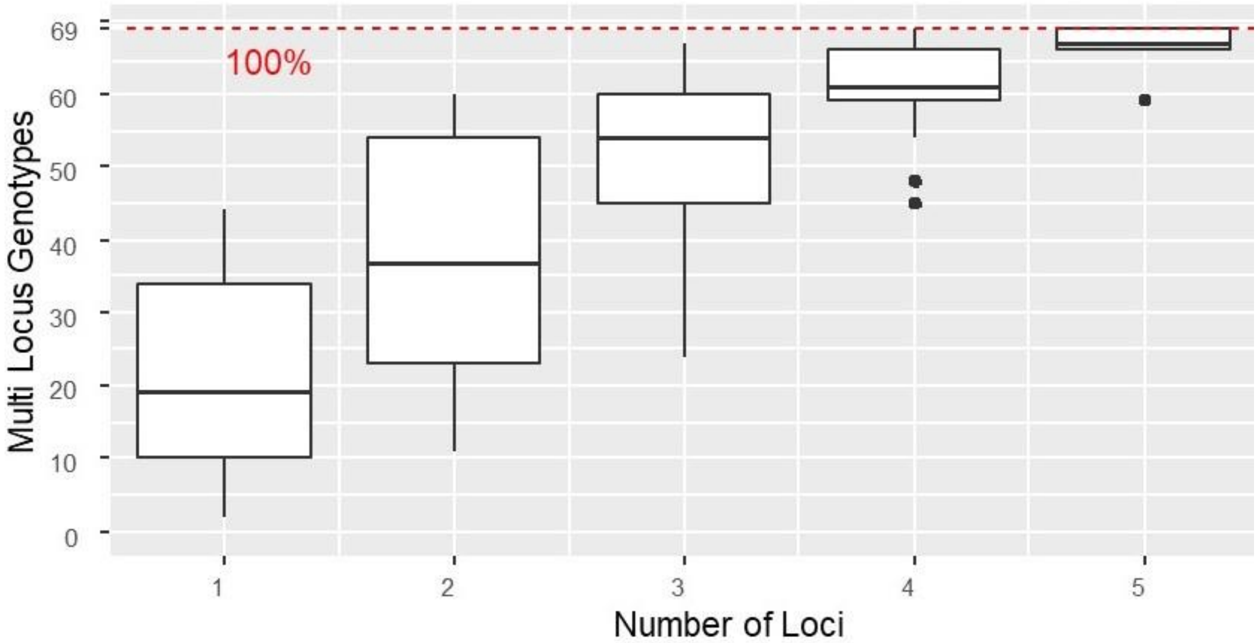

Figure S2. Genotype accumulation curve. For each boxplot, the six loci were randomly sampled 1000 times. A plateau was reached with the six loci.

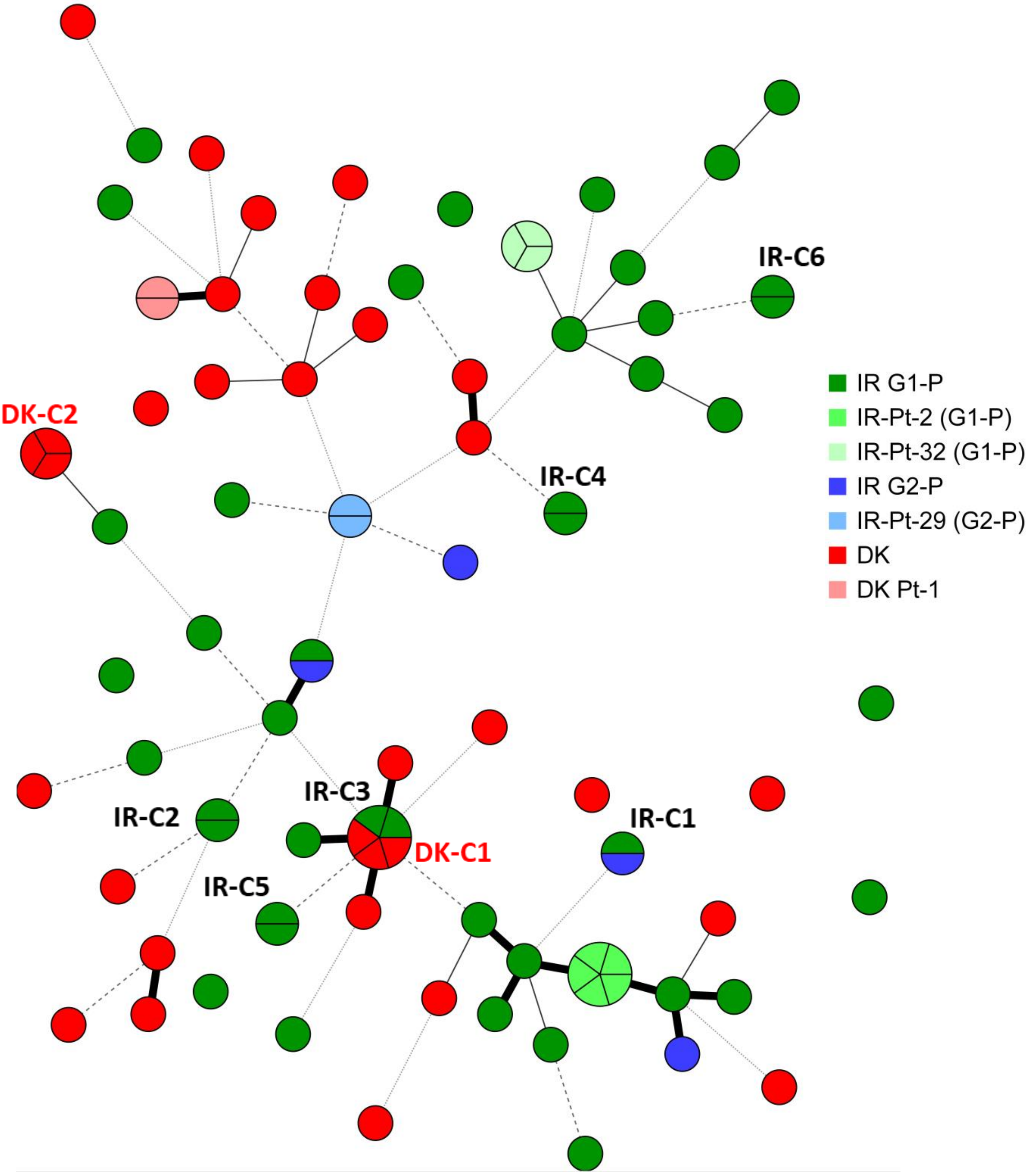

Figure S3. Microsatellite typing [2] of Danish and Iranian isolates visualized as a minimum spanning tree in BioNumerics v7.6. Green colors represent *C. parapsilosis* isolates belonging to AFLP cluster G1-P, blue is AFLP cluster G2-P strains and red are Danish isolates. There are six Iranian clusters and two Danish clusters involving different patients. No apparent correlation between AFLP fingerprints and microsatellite genotypes was observed.

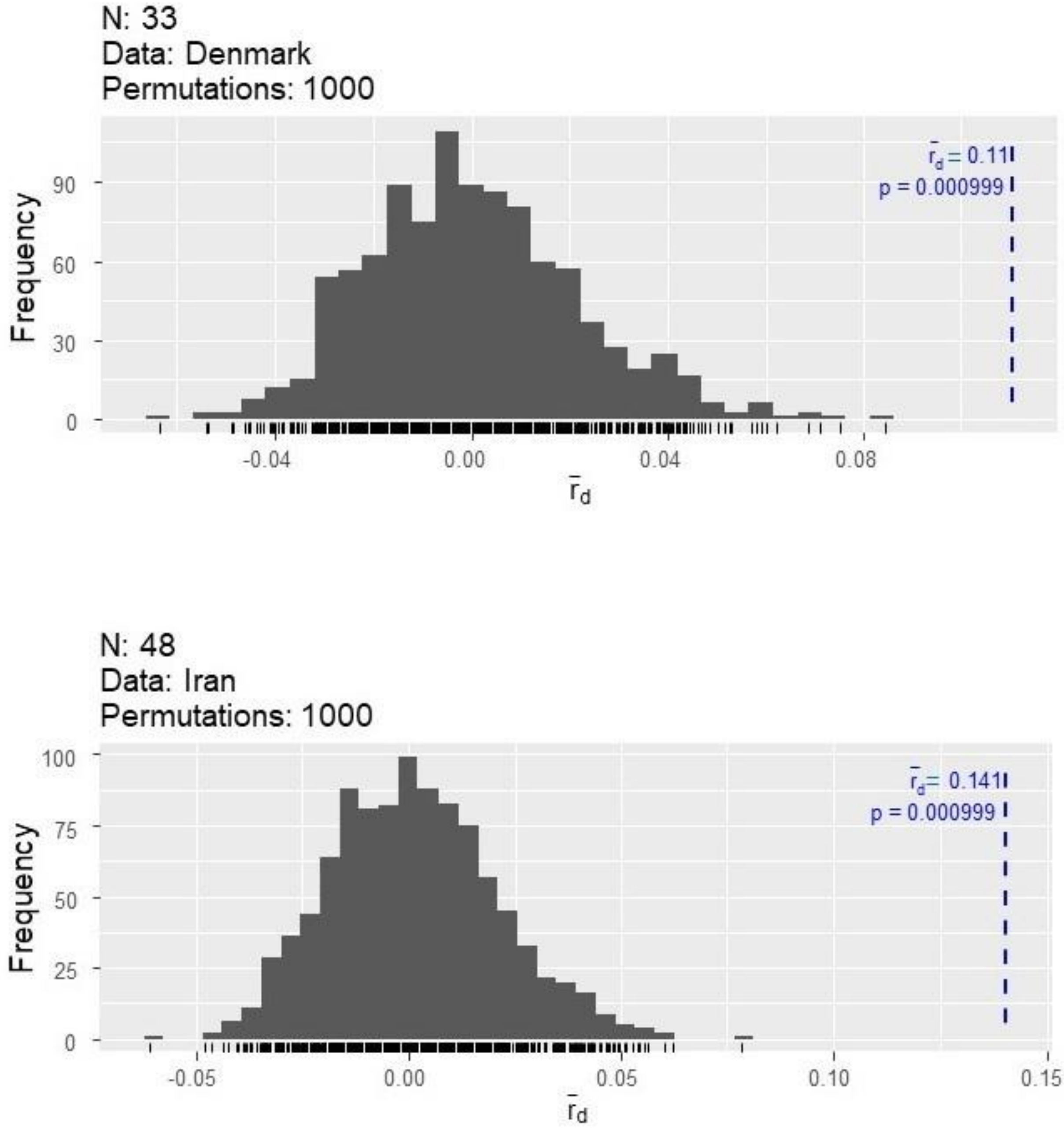

Figure S4. Test for linkage disequilibrium among the loci from the Danish and Iranian isolates of *Candida parapsilosis*. The $\bar{r}_d$ index accounts for the number of loci sampled, and is less biased than Index of Association. The low *P* values reject the null hypothesis of no linkage among markers.

1. Arastehfar A, Khodavaisy S, Daneshnia F, Najafzadeh M-J, Mahmoudi S, Charsizadeh A, et al. Molecular Identification, Genotypic Diversity, Antifungal Susceptibility, and Clinical Outcomes of Infections Caused by Clinically Underrated Yeasts, Candida orthopsilosis, and Candida metapsilosis: An Iranian Multicenter Study (2014–2019). *Front Cell Infect Microbiol*. **2019**;9(July). DOI: 10.3389/fcimb.2019.00264
2. Diab-Elschahawi M, Forstner C, Hagen F, Meis JF, Lassnig AM, Presterl E, et al. Microsatellite genotyping clarified conspicuous accumulation of Candida parapsilosis at a cardio-thoracic surgery intensive care unit. *J Clin Microbiol*. **2012**;50(August):3422–6. DOI: 10.1128/JCM.01179-12
