# Supplementary material for: Candidemia among Hospitalized Pediatric Patients Caused by Several Clonal Lineages of Candida parapsilosis
Source: J Fungi (Basel). 2022 Feb 12;8(2):183. doi: 10.3390/jof8020183 (PMC8880282; doi:10.3390/jof8020183)
Supplement: Supplementary file 1 [file jof-08-00183-s001.zip › final Cparapsilosis typing - Table S1.pdf]

Supplementary Table S1. Clinical data of patients included in this study.

Patient No	Age/sex	Weight (kg)	Ward	Underlying disease	Admission date	*Candida* isolation date	Isolation day after admission	CVC	TPN	Intubation and MV	Foley catheter	surgery	colonization	Neutropenia	Corticosteroid therapy	chemotherapy	*Candida* species	Duration of antifungal therapy	Outcome	Strain No
1	4y/F	16.2	Surgery	Genetic disorder	03.06.2017	09.06.2017	day 6	-	-	-	+	+	-	-	-	-	*C. parap*	15 d	Alive	811
2	12d/M	3.1	NICU-OH	CHD	23.05.2017	23.05.2017	day 1	+	+	+	+	+	-	-	-	-	*C. parap*	20 d	Died	797, 799, 800, 801 & 802
3	11m/F	10.7	Abd. surgery	Foreign body ingestion	07.06.2017	12.06.2017	day 5	+	+	+	-	+	+	_	_	_	*C. parap*	15 d	Died	796
4	7m/M	6.45	ID	Immunodeficiency	08.05.2017	25.02.2017	day 17	_	_	_	_	_	+	+	_	_	*C. parap*	21 d	Alive	795
5	1y/M	7.2	BMT	SCID (HSCT)	04.05.2017	25.05.2017	day 21	+	_	+	+	+	+	_	+	_	*C. parap*	Under treatment	Died	784
6	3d/M	2.9	NICU	Ichthyosis vulgaris	19.03.2017	31.03.2017	day 12	+	+	+	_	_	+	_	_	_	*C. parap*	12 d	Died	770
7	10y/F	30.0	PICU	Wolfram syndrome	01.04.2017	16.04.2017	day 15	+	_	_	+	+	_	_	_	_	*C. parap*	7 d under treatment	Died	766
8	8y/F	21.0	PICU	Cystic fibrosis	31.01.2017	04.02.2017	day 4	+	_	+	_	_	+	_	_	_	*C. parap*	12 d	Alive	748
9	3y/M	13.8	PICU	ALL	02.02.2017	25.02.2017	day 23	+	_	_	_	_	_	+	_	+	*C. parap*	21 d	Alive	747
10	5y/F	16.5	PICU	Metabolic disease	04.01.2017	19.04.2017	day 16	+	_	+	_	+	_	_	_	_	*C. parap*	14 d	Alive	740
11	16d/M	1.4	NICU	Prematurity	22.11.2016	29.11.2016	day 7	+	+	+	_	_	_	_	_	_	*C. parap*	10 d	Alive	723

12	2y/F	11.1	PICU	Metabolic disease	01.10.2016	01.11.2016	day 30	+	+	_	_	_	_	_	_	_	*C. parap*	7 d under treatment	Died	706
13	4y/F	14.5	EICU	Metabolic disease	17.09.2016	28.06.2016	day 11	+	+	+	+	_	+	_	_	_	*C. parap*	3 d under treatment	Died	685
14	2y/F	11.9	EICU	ALL	25.06.2016	12.07.2016	day 17	+	+	+	_	_	_	+	_	+	*C. parap*	14 d	Alive	650
15	7y/M	23.0	PICU	Lymphoma	20.06.2016	15.07.2016	day 45	+	_	+	+	_	_	_	_	_	*C. ortho*	17 d	Alive	618
16	1y/F	7.2	Immunology	Immunodeficiency	15.06.2016	07.07.2016	day 22	+	_	+	_	_	_	+	_	_	*C. ortho*	14 d	Alive	617
17	1m/M	2.2	NICU	Prematurity	01.06.2016	16.06.2016	day 15	+	+	+	_	_	_	_	_	_	*C. ortho*	15 d	Alive	606
18	5m/F	3.5	EICU	prematurity	16.05.2016	12.07.2016	day 57	+	+	+	_	+	_	_	_	_	*C. parap*	12 d under treatment	Died	602
19	2m/M	3.95	PICU	Antenatal Hydronephrosis	30.01.2016	08.02.2016	day 9	+	_	_	+	+	+	_	_	_	*C. parap*	14 d	Alive	529
20	8y/M	21.0	PICU	Hyper IgM syndrome	21.11.2015	26.11.2015	day 5	+	_	+	_	_	+	+	_	_	***C. ortho***	18 d	Alive	496
21	18d/M	1.95	NICU	Prematurity	16.09.2015	04.10.2015	day 16	+	+	+	_	+*	_	_	_	_	*C. parap*	12 d	Alive	427
22	4m/M	3.8	PICU	Prematurity	05.07.2015	07.07.2015	day 3	+	+	+	_	_	_	_	+	_	*C. parap*	3 d under treatment	Died	371
23	2d/M	3.1	NICU	Epidermolysis bullosa	26.06.2015	09.07.2015	day 13	+	+	_	_	_	+	_	_	_	*C. parap*	14 d	Alive	363

24	20d/F	1.9	PICU	Prematurity	25.06.2015	15.06.2015	day 25	+	+	+	_	_	_	_	_	_	*C. parap*	3 d under treatment	Died	362
25	3m/M	5.7	PICU	VACTERL	26.06.2015	26.07.2015	day 30	+	_	+	_	+	+	_	_	_	*C. parap*	8 d under treatment	Died	360
26	5d/F	3.4	NICU	AML	18.05.2015	02.06.2015	day 14	+	+	_	_	_	_	+	_	+	*C. parap*	6 d under treatment	Died	359
27	1y/M	7.9	PICU	Prematurity	25.05.2015	16.06.2015	day 22	+	+	+	_	+	+	_	_	_	*C. parap*	Under treatment	Died	351
28	11m/M	8.2	Immunology	SCID	11.05.2015	13.06.2015	day 43	+	+	_	_	_	+	+	_	_	*C. parap*	15 d	Alive	341
29	16d/M	1.73	NICU	Prematurity	21.04.2015	22.04.2015	day 2	+	_	+	+	+*	+	_	_	_	*C. parap*	AMB 3 d CAS 10 d	Alive	293 & 296
30	10m/F	7.0	CICU	CHD	24.04.2015	25.04.2015	day 2	+	_	+	_	+	_	_	_	_	*C. parap*	7 d under treatment	Died	295
31	2y/M	13.0	EICU	Brain tumor	23.04.2015	30.5.2015	day 37	+	+	+	+	+	_	_	+	+	*C. parap*	7 d under treatment	Died	292
32	4y/M	13.5	PICU	Hyper IgE syndrome	03.03.2015	24.03.2015	day 21	+	_	+	+	_	+	_	_	_	*C. parap*	AMB 3 d CAS 10 d	Alive	229, 254 & 291
33	1y/F	11.7	PICU	Charcot-Marie-tooth disease	11.02.2015	29.02.2015	day 18	+	_	+	_	+	+	_	_	_	*C. parap*	6 d under treatment	Died	281
34	1y/M	12.2	PICU	Encephalopathy following MMR vaccine	10.02.2015	05.03.2015	day 23	_	+	_	+	_	+	-	+	-	*C. parap*	21 d	Died	265
35	4m/M	4.8	GI	Cystic fibrosis	03.02.2015	15.02.2015	day 12	+	-	+	_	_	+	_	_	_	*C. parap*	3 d under treatment	Died	231
36	1m/F	4.2	CICU	Tuberous Sclerosis	28.02.2015	06.03.2015	day 6	+	_	+	_	+	+	_	_	_	*C. parap*	10 d	Alive	223

37	2y/F	11.9	ID	Intramedullary spinal cord tumor	04.02.2015	12.02.2015	day 8	+	_	_	_	+	_	+	_	+	*C. parap*	14 d	Alive	194
38	3m/F	3.0	ID	Prematurity	03.01.2015	20.01.2015	day 12	+	+	+	_	_	+	_	_	_	*C. parap*	10 d	Alive	162
39	3y/F	13.5	PICU	ALL	24.12.2014	27.01.2015	day 32	+	_	+	_	+*	_	+	+	+	*C. ortho*	12 d	Alive	187
40	3m/F	5,85	Neurology	Neuroblastoma	20.10.2014	17.11.2014	day 28	_	+	_	+	-	_	+	_	+	*C. parap*	8 d under treatment	Died	125
41	6y/F	17.5	CICU	CHD	24.11.2014	13.12.2014	day 19	+	+	+	+	+	+	_	_	_	*C. parap*	3 d under treatment	Died	117
42	4m/M	6.15	ID	Post meningitis encephalopathy	06.11.2014	13.11.2014	day 7	_	+	_	_	_	+	_	_	_	*C. parap*	14 d	Alive	104
43	3m/M	2.75	PICU	Metabolic disease	06.11.2014	15.11.2014	day 9	+	+	+	_	+	_	+	+	_	*C. parap*	10 d	Died	103, 185 & 241
44	1y/M	12.3	Neurology	Encephalopathy following MMR vaccine	29.09.2014	02.10.2014	day 3	+	_	+	_	+	_	_	_	_	*C. parap*	-	Died	83
45	5m/F	4.1	EICU	Prematurity	09.10.2014	29.11.2014	day 51	+	+	+	_	+	_	_	+	_	*C. parap*	4 d under treatment	Died	82
46	1y/F	9.85	GI	Prematurity	03.10.2014	12.10.2014	day 9	+	+	_	_	+	+	_	_	_	*C. parap*	14 d	Alive	77
47	28d/F	2.9	NICU	Metabolic disease/ prematurity	24.09.2014	15.10.2014	day 22	+	+	+	_	_	_	_	_	_	*C. parap*	12 d	Alive	69

Abbreviations: CVC: central venous catheter, TPN: total parenteral nutrition, MV: Mechanical ventilation, y: year, m: months, d, days, M: male, F: female, Abd.: Abdominal, ICU: Intensive care unit, PICU: Pediatric ICU, NICU: Neonatal ICU, NICU-OH: NICU Open Heart Surgery, CICU: Cardiac ICU, EICU: Emergency ICU, ID: Infectious diseases, GI: Gastroenterology, BMT: Bone marrow transplant, CHD: Coronary Heart Disease., AMB: Amphotericin B, CAS: Caspofungin,
