# Supplementary material for: Candidemia among Hospitalized Pediatric Patients Caused by Several Clonal Lineages of Candida parapsilosis
Source: J Fungi (Basel). 2022 Feb 12;8(2):183. doi: 10.3390/jof8020183 (PMC8880282; doi:10.3390/jof8020183)
Supplement: Supplementary file 1 [file jof-08-00183-s001.zip › final Cparapsilosis typing - Table S2.pdf]

**Supplementary Table S2**. *Candida parapsilosis* microsatellite data, based on six diploid tandem repeat markers as previously described [1]. Number of tandem repeats (TR) were calculated and specific fragment sizes corresponding to a number of repeats for each marker was indicated.

| | **Tri-nucleotide microsatellite markers** | | | | | | **Hexa-nucleotide microsatellite markers** | | | | | |
|---|---|---|---|---|---|---|---|---|---|---|---|---|
| | **3A_I** | **3A_II** | **3B_I** | **3B_II** | **3C_I** | **3C_II** | **6A_I** | **6A_II** | **6B_I** | **6B_II** | **6C_I** | **6C_II** |
| **DK-ID** | 194.4 = 24 TR | | 202.4 = 29 TR | | 190.4 = 26 TR | | 161.2 = 8 TR | | 132.4 = 8 TR | | 122.8 = 7 TR | |
| DK1 | 24 | 24 | 29 | 29 | 41 | 41 | 8 | 8 | 8 | 8 | 7 | 7 |
| DK10 | 24 | 24 | 29 | 31 | 41 | 41 | 8 | 8 | 8 | 8 | 7 | 7 |
| DK11 | 18 | 18 | 40 | 40 | 39 | 39 | 12 | 12 | 8 | 8 | 7 | 7 |
| DK12 | 23 | 25 | 26 | 32 | 26 | 26 | 12 | 12 | 8 | 8 | 7 | 7 |
| DK13 | 24 | 24 | 29 | 29 | 41 | 41 | 8 | 8 | 8 | 8 | 7 | 7 |
| DK14 | 23 | 25 | 26 | 32 | 26 | 26 | 12 | 12 | 8 | 8 | 7 | 7 |
| DK15 | 24 | 24 | 29 | 29 | 41 | 41 | 8 | 9 | 8 | 8 | 7 | 7 |
| DK16 | 25 | 26 | 38 | 38 | 37 | 38 | 8 | 8 | 8 | 8 | 7 | 7 |
| DK17 | 24 | 24 | 23 | 23 | 25 | 25 | 12 | 12 | 9 | 9 | 9 | 9 |
| DK18 | 24 | 24 | 34 | 34 | 25 | 26 | 13 | 13 | 8 | 8 | 7 | 7 |
| DK19 | 24 | 25 | 29 | 30 | 26 | 26 | 17 | 17 | 8 | 8 | 7 | 7 |
| DK2 | 18 | 25 | 40 | 40 | 40 | 46 | 12 | 12 | 8 | 8 | 7 | 7 |
| DK20 | 25 | 31 | 34 | 41 | 46 | 46 | 12 | 13 | 8 | 8 | 7 | 7 |
| DK21 | 18 | 25 | 28 | 40 | 46 | 46 | 12 | 12 | 8 | 8 | 7 | 7 |
| DK22 | 24 | 24 | 39 | 44 | 38 | 38 | 15 | 15 | 8 | 8 | 7 | 13 |
| DK23 | 25 | 25 | 15 | 29 | 43 | 43 | 12 | 12 | 8 | 8 | 7 | 7 |
| DK24 | 24 | 26 | 30 | 30 | 27 | 39 | 12 | 12 | 8 | 8 | 12 | 12 |
| DK25 | 24 | 24 | 34 | 38 | 40 | 42 | 8 | 8 | 8 | 8 | 7 | 7 |
| DK26 | 23 | 25 | 26 | 32 | 26 | 26 | 12 | 12 | 8 | 8 | 7 | 7 |
| DK27 | 24 | 24 | 29 | 29 | 41 | 41 | 8 | 8 | 8 | 8 | 7 | 7 |
| DK28 | 18 | 25 | 28 | 45 | 46 | 46 | 12 | 13 | 8 | 8 | 7 | 7 |
| DK29 | 24 | 24 | 29 | 35 | 43 | 43 | 8 | 8 | 8 | 8 | 7 | 10 |
| DK3 | 21 | 33 | 29 | 29 | 40 | 40 | 8 | 8 | 9 | 9 | 7 | 7 |
| DK30 | 18 | 26 | 28 | 46 | 46 | 46 | 12 | 12 | 8 | 8 | 7 | 7 |
| DK31 | 24 | 25 | 35 | 35 | 26 | 33 | 13 | 17 | 8 | 8 | 7 | 7 |
| DK32 | 25 | 32 | 34 | 41 | 46 | 46 | 12 | 12 | 8 | 8 | 7 | 7 |
| DK33 | 26 | 26 | 33 | 33 | 57 | 57 | 12 | 12 | 8 | 8 | 7 | 7 |
| DK34 | 25 | 32 | 34 | 41 | 46 | 46 | 12 | 12 | 8 | 8 | 7 | 7 |
| DK4 | 25 | 25 | 29 | 39 | 42 | 42 | 8 | 8 | 8 | 8 | 7 | 7 |
| DK5 | 24 | 25 | 35 | 35 | 26 | 26 | 13 | 17 | 8 | 8 | 7 | 7 |
| DK6 | 25 | 25 | 34 | 41 | 46 | 46 | 12 | 12 | 8 | 8 | 7 | 7 |
| DK7 | 24 | 25 | 29 | 35 | 40 | 40 | 8 | 8 | 8 | 8 | 7 | 7 |
| DK8 | 24 | 25 | 30 | 30 | 27 | 39 | 12 | 12 | 8 | 8 | 12 | 12 |
| DK9 | 13 | 24 | 32 | 32 | 27 | 27 | 12 | 12 | 8 | 8 | 7 | 7 |
| **IR-ID** | | | | | | | | | | | | |

| | | | | | | | | | | | | |
|---|---|---|---|---|---|---|---|---|---|---|---|---|
| 241 | 21 | 21 | 39 | 39 | 27 | 27 | 12 | 15 | 8 | 8 | 7 | 7 |
| 254 | 24 | 25 | 35 | 39 | 27 | 27 | 9 | 12 | 8 | 8 | 7 | 7 |
| 229 | 24 | 25 | 35 | 39 | 27 | 27 | 9 | 12 | 8 | 8 | 7 | 7 |
| 231 | 24 | 24 | 29 | 29 | 26 | 26 | 13 | 13 | 8 | 8 | 7 | 7 |
| 223 | 24 | 24 | 29 | 29 | 26 | 26 | 13 | 13 | 8 | 8 | 7 | 7 |
| 194 | 24 | 24 | 29 | 29 | 41 | 41 | 8 | 8 | 8 | 8 | 7 | 7 |
| 185 | 21 | 21 | 39 | 39 | 27 | 27 | 9 | 9 | 8 | 8 | 7 | 7 |
| 162 | 24 | 24 | 24 | 24 | 26 | 26 | 12 | 12 | 8 | 8 | 7 | 7 |
| 147 | 25 | 25 | 30 | 30 | 18 | 18 | 12 | 12 | 8 | 8 | 12 | 12 |
| 125 | 24 | 25 | 28 | 28 | 55 | 55 | 12 | 12 | 8 | 8 | 7 | 13 |
| 117 | 24 | 24 | 25 | 29 | 45 | 45 | 8 | 8 | 8 | 8 | 7 | 7 |
| 104 | 25 | 25 | 30 | 30 | 18 | 18 | 12 | 12 | 8 | 8 | 12 | 12 |
| 103 | 24 | 24 | 25 | 29 | 45 | 45 | 8 | 8 | 8 | 8 | 7 | 7 |
| 83 | 25 | 25 | 25 | 26 | 28 | 28 | 12 | 12 | 8 | 8 | 7 | 7 |
| 82 | 24 | 24 | 29 | 29 | 26 | 32 | 12 | 12 | 8 | 8 | 7 | 7 |
| 77 | 24 | 24 | 27 | 27 | 31 | 31 | 12 | 12 | 8 | 8 | 4 | 7 |
| 69 | 24 | 24 | 36 | 37 | 40 | 40 | 8 | 8 | 8 | 8 | 7 | 7 |
| 186 | 26 | 26 | 34 | 34 | 56 | 56 | 12 | 12 | 8 | 8 | 7 | 7 |
| 265 | 24 | 25 | 28 | 28 | 27 | 56 | 12 | 12 | 8 | 8 | 13 | 13 |
| 281 | 23 | 25 | 30 | 30 | 27 | 27 | 9 | 9 | 8 | 8 | 7 | 7 |
| 291 | 24 | 25 | 35 | 39 | 27 | 27 | 9 | 12 | 8 | 8 | 7 | 7 |
| 292 | 17 | 32 | 22 | 27 | 26 | 26 | 13 | 13 | 8 | 8 | 7 | 9 |
| 293 | 24 | 25 | 28 | 33 | 56 | 66 | 12 | 12 | 8 | 8 | 7 | 7 |
| 295 | 25 | 33 | 30 | 30 | 27 | 34 | 12 | 12 | 8 | 8 | 7 | 7 |
| 296 | 24 | 25 | 28 | 33 | 56 | 66 | 12 | 12 | 8 | 8 | 13 | 17 |
| 341 | 24 | 25 | 35 | 36 | 45 | 46 | 8 | 8 | 8 | 8 | 7 | 13 |
| 351 | 25 | 26 | 30 | 30 | 32 | 32 | 12 | 12 | 8 | 8 | 3 | 3 |
| 359 | 24 | 25 | 30 | 30 | 27 | 34 | 9 | 14 | 8 | 8 | 7 | 7 |
| 360 | 26 | 26 | 30 | 30 | 27 | 27 | 9 | 9 | 8 | 8 | 7 | 7 |
| 363 | 24 | 24 | 38 | 38 | 40 | 40 | 8 | 8 | 8 | 8 | 7 | 7 |
| 371 | 24 | 25 | 26 | 26 | 49 | 49 | 8 | 8 | 8 | 8 | 7 | 7 |
| 427 | 24 | 24 | 50 | 54 | 27 | 27 | 12 | 12 | 8 | 8 | 7 | 7 |
| 529 | 24 | 26 | 30 | 30 | 27 | 27 | 9 | 14 | 8 | 8 | 7 | 7 |
| 582 | 24 | 24 | 37 | 37 | 49 | 49 | 8 | 8 | 8 | 8 | 7 | 7 |
| 685 | 24 | 24 | 38 | 49 | 40 | 40 | 8 | 8 | 8 | 8 | 7 | 7 |
| 706 | 24 | 24 | 38 | 38 | 40 | 46 | 8 | 8 | 8 | 8 | 7 | 7 |
| 723 | 24 | 24 | 37 | 37 | 40 | 40 | 8 | 8 | 8 | 8 | 7 | 7 |
| 747 | 24 | 24 | 29 | 34 | 41 | 41 | 8 | 8 | 8 | 8 | 7 | 7 |
| 748 | 24 | 25 | 30 | 30 | 27 | 27 | 9 | 12 | 8 | 8 | 7 | 7 |
| 766 | 25 | 25 | 14 | 30 | 33 | 34 | 9 | 14 | 8 | 8 | 7 | 7 |
| 770 | 24 | 24 | 29 | 37 | 40 | 40 | 8 | 8 | 8 | 8 | 7 | 7 |
| 784 | 24 | 24 | 29 | 29 | 41 | 41 | 8 | 8 | 8 | 8 | 7 | 7 |
| 795 | 25 | 25 | 14 | 30 | 33 | 34 | 9 | 14 | 8 | 8 | 7 | 7 |

| | | | | | | | | | | | | |
|---|---|---|---|---|---|---|---|---|---|---|---|---|
| 796 | 25 | 26 | 30 | 30 | 27 | 40 | 12 | 14 | 8 | 8 | 12 | 12 |
| 797 | 24 | 24 | 37 | 38 | 40 | 40 | 8 | 8 | 8 | 8 | 7 | 7 |
| 799 | 24 | 24 | 37 | 38 | 40 | 40 | 8 | 8 | 8 | 8 | 7 | 7 |
| 800 | 24 | 24 | 37 | 38 | 40 | 40 | 8 | 8 | 8 | 8 | 7 | 7 |
| 801 | 24 | 24 | 37 | 38 | 40 | 40 | 8 | 8 | 8 | 8 | 7 | 7 |
| 802 | 24 | 24 | 37 | 38 | 40 | 40 | 8 | 8 | 8 | 8 | 7 | 7 |
| 650 | 24 | 31 | 23 | 37 | 48 | 48 | 8 | 8 | 8 | 8 | 7 | 7 |
| 811 | 24 | 24 | 29 | 33 | 26 | 32 | 12 | 12 | 8 | 8 | 7 | 7 |
| 749 | 24 | 24 | 29 | 33 | 26 | 32 | 12 | 12 | 8 | 8 | 7 | 7 |
| 740 | 21 | 24 | 30 | 31 | 41 | 55 | 8 | 8 | 8 | 8 | 7 | 8 |
| 602 | 24 | 31 | 23 | 37 | 48 | 48 | 8 | 8 | 8 | 8 | 7 | 7 |
| 711 | 23 | 25 | 25 | 25 | 26 | 26 | 12 | 12 | 8 | 8 | 7 | 7 |

1. Diab-Elschahawi M, Forstner C, Hagen F, Meis JF, Lassnig AM, Presterl E, et al. Microsatellite genotyping clarified conspicuous accumulation of *Candida parapsilosis* at a cardio-thoracic surgery intensive care unit. *J Clin Microbiol*. **2012**;50:3422–6. DOI: 10.1128/JCM.01179-12
