# Supplementary material for: Candidemia among Hospitalized Pediatric Patients Caused by Several Clonal Lineages of Candida parapsilosis
Source: J Fungi (Basel). 2022 Feb 12;8(2):183. doi: 10.3390/jof8020183 (PMC8880282; doi:10.3390/jof8020183)
Supplement: Supplementary file 1 [file jof-08-00183-s001.zip › final Cparapsilosis typing - Table S3.pdf]

Supplementary Table S3. Antifungal susceptibility data and Fks1 and Erg11 profiles.

Isolates #	Species	AFLP Genotype	MIC medians (range), mg/L					*ERG11* SNPs (amino acid substitution)	HS1 *FKS1*	HS2 *FKS2*	Details
			FLC	VRC	MIC	AND	AMB				
Multiple	*C. parapsilosis*	G1-P	0.5 (0.25–2)	≤0.03 (≤0.03)	2 (1-2)	1 (0.5-4)	0.125 (0.06–0.5)	WT	FLTLSLRDA	DWIRRYTL	35 isolates, 31 patients, 18 Alive, 13 died
650, 685, 811	*C. parapsilosis*	G2-P	1 (0.5–1)	≤0.03 (≤0.03)	1 (1-2)	1 (0.5-2)	0.125 (0.125)	WT	FLTLSLRDA	DWIRRYTL	3 isolates, 3 patients, 2 Alive, 1 died
82	*C. parapsilosis*	G1-P	0.5	≤0.03	1	1	0.125	T905C (I302T)	FLTLSLRDA	DWIRRYTL	Died
362	*C. parapsilosis*	G1-P	0.25	≤0.03	1	1	0.25	G397T (D133Y)	FLTLSLRDA	DWIRRYTL	Died
125	*C. parapsilosis*	G1-P	0.5	≤0.03	2	2	0.125	C748A (Q250K), G1193T (R398I)	FLTLSLRDA	DWIRRYTL	Died
254* & 291*	*C. parapsilosis*	G1-P	0.5–1	≤0.03	1	0.5	0.125	WT	FLTLSLRDA	DWIRRYTL	Alive
229*	*C. parapsilosis*	G1-P	0.5	≤0.03	2	0.5	0.25	G397T (D133Y)	FLTLSLRDA	DWIRRYTL	Alive
185**, 241**	*C. parapsilosis*	G1-P	0.5	≤0.03	2	2	0.125	WT	FLTLSLRDA	DWIRRYTL	Died
103**	*C. parapsilosis*	G1-P	1	≤0.03	2	1	0.125	C748A (Q250K)	FLTLSLRDA	DWIRRYTL	Died
293***, 296***	*C. parapsilosis*	G2-P	0.25–0.5	≤0.03	1-2	1–2	0.125–0.25	G1193T (R398I)	FLTLSLRDA	DWIRRYTL	Alive
265	*C. parapsilosis*	G2-P	1	≤0.03	2	2	0.25	G1193T (R398I)	FLTLSLRDA	DWIRRYTL	Died
147, 187, 496	*C. orthopsilosis*	G1-Orth	0.25 (0.25–0.5)	≤0.03 (≤0.03)	0.5 (0.5)	0.5 (0.5)	0.06 (0.06–0.125)	WT	FLTLSLRDA	DWVRRYTL	3 isolates, 2 patients, all alive
606, 617, 618	*C. orthopsilosis*	G2-Orth	0.5 (0.25–2)	≤0.03 (≤0.03)	0.5 (0.5)	0.5 (0.25–0.5)	0.06 (0.06–0.125)	WT	FLTLSLRDA	DWVRRYTL	3 isolates, 3 patients, all alive

WT: Wild-type. SNP: single nucleotide polymorphism. All of the *ERG11* sequences harbored the silent T591C mutation

*. **. ***. and **** These isolates were recovered from a single patient.

G1-P and G2-P represent genotypes 1 and 2 of *C. parapsilosis*. while G1orth and G2orth represent genotypes 1 and 2 of *C. orthopsilosis*.

Deposition of isolates and sequence data

Six *C. orthopsilosis* isolates obtained from this study were deposited in the CBS yeast culture collection hosted at the Westerdijk Fungal Biodiversity Institute, Utrecht, the Netherlands, with the accession numbers CBS 15856–CBS 15861. Sequences obtained for *ERG11* (MK521929–MK521979) and HS1 and HS2 of *FKS1* (MK522274–MK522386) were deposited in GenBank.
